# Mature compost enhanced the harmlessness level in co-composting swine manure and carcasses in large-scale silo reactors

**DOI:** 10.3389/fmicb.2024.1494332

**Published:** 2024-11-13

**Authors:** Ziwei Jiao, Liping Zhang, Ake Zhang, Ruoqi Li, Kui Zhang, Zhen Wu, Zitong Kang, Yuquan Wei, Longli Zhang, Yue Wang, Xiong Shi, Ji Li

**Affiliations:** ^1^College of Biological Science and Technology, Yili Normal University, Yining, China; ^2^College of Resources and Environmental Science, Beijing Key Laboratory of Biodiversity and Organic Farming, China Agricultural University, Beijing, China; ^3^Fuyang Agricultural Science Academy, Fuyang, Anhui, China; ^4^College of Agricultural Science and Engineering, Hohai University, Nanjing, Jiangsu, China; ^5^Beijing VOTO Biotech Co., Ltd., Beijing, China; ^6^Yangtze Eco-Environment Engineering Research Center, China Three Gorges Corporation, Wuhan, China

**Keywords:** full-scale composting, silo reactor, swine manure, bacterial community, potential pathogen, mature compost

## Abstract

This study aimed to investigate the impact of incorporating mature compost on the harmlessness and maturity level of composting from swine manure and carcasses from industrialized pig farms in continuously running large-scale silo reactor systems. The potential human or animal bacterial pathogens and core bacterial community in composting were analyzed by high-throughput sequencing of 16S rRNA gene amplicons. The results showed that the addition of mature compost in the GD group significantly increased the temperature of all depths, the accumulated temperature of compost, and the germination index (75.43%) compared to that in the HN group without mature compost. High-throughput sequencing revealed that the dominated genera in GD were *Ureibacillus, Lactobacillus*, *Corynebacterium*, *Staphylococcus*, and *Jeotgalicoccus*, and the addition of mature compost could significantly increase the relative abundance of *Ureibacillus* (16.82%) that was associated with the biodegradation of organics. A total of 421 potential bacterial pathogens were detected, and the dominated genera of pathogens were *Streptococcus*, *Staphylococcus*, and *Anaerococcus*. The potential pathogen in the GD group with mature compost was reduced from 7.16 to 0.77%, which was significantly lower than that (2.97%) in the HN group. Together, these findings revealed that mature compost addition in large-scale reactor composting could accelerate the harmless and humification process, providing an effective and environmentally friendly scheme to deal with the main organic wastes in intensive pig farms.

## 1 Introduction

According to the National Bureau of Statistics in China, pork production was 55.41 million tons in 2022, which was 77.4% higher than beef and 27.9% higher than lamb. The high demand for pork in China due to a longstanding food culture spanning thousands of years leads to a large amount of pig farming and swine manure generation (Wang et al., 2021). Nevertheless, inadequate management of swine manure can result in serious environmental issues, such as nutrient leaching, pathogen contamination, and pollutant emissions (Li et al., 2023). On the other hand, pig carcasses, categorized as part of piggery waste, are deemed unsuitable for slaughter at commercial abattoirs, and proper disposal of carcasses is crucial for environmental sustainability, especially during widespread disease outbreaks such as the African Swine Fever epidemic and the COVID-19 pandemic (Ki et al., 2018). Given the potential nutrient resources present in these piggery wastes, composting emerges as a viable economic and environmental strategy for recycling such materials, through which organic components are biologically degraded and stabilized by microbial communities (Wei et al., 2019). The material can be finally converted into organic fertilizer or soil conditioner, realizing the resource utilization of organic wastes (Chen et al., 2023).

Microbial colonization and metabolism are critical for the fate and efficiency of the composting process, whose activities produce heat in piles, especially in the thermophilic phase, and help to eliminate pathogenic microorganisms (Wilkinson, 2007). *C*/*N* ratio is one of the important indicators at the initial of pig manure composting, which can be regulated by bulking materials such as straws, sawdust, and corn stalks to improve microbial activities for composting research at the lab scale (Zhang et al., 2020). However, the utilization of bulking materials in composting (1) can increase the volume of raw materials (decrease the volume of pig manure to be treated) due to their low density; (2) results in elevated composting costs, including transportation expenses and the procurement costs of bulking materials; and (3) possibly extends the composting period due to the refractory property of lignocellulose (Sun et al., 2016; Lu et al., 2020). Consequently, many large-scale composting facilities in China may opt to conduct composting operations without auxiliary materials, which cut down composting production costs. Paradoxically, this is difficult to ensure the fermentation efficiency of microorganisms, which may affect the quality of the compost products. Until now, there are a limited number of studies available on the disposal of swine manure and carcasses through large-scale composting, especially focusing on the level of harmlessness and the effect of potential pathogens.

The silo reactor is extensively utilized for composting factories in China, particularly in large-scale farms, which have different features from other contained, or in-vessel, composting methods. Zhang et al. (2016) found that the temperature of all layers in silo reactors could remain over 50°C until the end of composting. The cylindrical structure of silo reactors was found to facilitate the airflow stack effect, contributing to the maintenance of a high temperature and the reduction of the composting period through enhanced moisture removal (Liu et al., 2020). The livestock industry has a significant demand for the harmless treatment of swine manure and carcasses, and co-composting is gaining increasing attention (Avidov et al., 2021; Wang et al., 2024). Compared to swine manure, swine carcasses have relatively high moisture contents, and low *C*/*N* and porosity, which may limit microbial aerobic metabolism, compost warming, and even accumulate the ratio of anaerobic microbial community (Won et al., 2016). Wang and Akdeniz (2023) found that co-composting poultry carcasses with cow manure increases core compost temperatures by approximately 3°C. Liu et al. (2016) found that amino acids hydrolyzed from animal carcasses are a good additive for the production of bio-organic fertilizer, improving the compost quality. According to our previous research on Chinese organic fertilizer enterprises, it was discovered that there were 629 potential pathogens present in compost products, accounting for an average of 1.21% of the total 16S rRNA gene (Wang et al., 2020). While several studies have investigated the microbial activity in co-composting swine manure and carcasses with bulking materials on, few have examined the detailed monitoring of microbial community succession and pathogen reduction of co-composting swine carcasses and manure in a large-scale setting without bulking materials.

Due to the large size of swine carcasses, composting can last several months or even years for complete degradation. The composting of carcasses differs from other types of substrates in various physicochemical properties (Wilkinson, 2007). Studies have shown that temperature and microbial activity may be the key factors in destroying viruses during the composting process (Vitosh-Sillman et al., 2017). Mature compost inoculation appears to meet these needs. It is widely recognized that inoculating external microbial agents into the composting process can alter bacterial community succession, enhance enzyme activities, and improve the temperature (Wei et al., 2018; Wu et al., 2024). However, commercial microbial inoculum is expensive and may compete with natural microorganisms in raw compost materials (Wang et al., 2022). Mature compost, rich in diverse microorganisms and enzyme activities (e.g., peroxidase, arylsulfatase, and urease contents during the mesophilic phase), can be used to inoculate indigenous microbes and use the swine carcasses substrate (Ma et al., 2019). It was found that mature compost addition increased the abundance of *Ureibacillus*, *Lysinibacillus*, *Limochordaceae*, and *Tepidimicrobium* by creating a suitable temperature environment, which improved carbohydrate metabolism functions and facilitated the decomposition of organic matter (Wang et al., 2024). Moreover, mature compost, low moisture, and porous structure can improve the raw material structure and make it a cost-effective alternative to bulking materials (Li et al., 2023; Yang and Zhang, 2022). Therefore, mature compost inoculation could accelerate the inactivation of pathogens, the degradation of carcasses, and the maturation of the compost pile humification process. Unlike many studies that focus on synthetic or commercial inoculants, this study investigates the potential of mature compost as a natural and cost-effective inoculum to enhance microbial succession and compost quality during large-scale composting. This approach minimizes costs and provides a sustainable alternative to commercial products.

Previous studies on composting have often overlooked the co-composting of large swine carcasses and manure in industrial contexts, especially regarding the detailed monitoring of microbial community succession and pathogen reduction. This research fills this gap by focusing on the large-scale application of silo reactors, which provides insights into the practical challenges and solutions for industrial composting in China’s pig farming sector.

Consequently, this study aims to explore (1) the effects of adding mature compost on temperature and maturity during the composting process of swine manure and carcasses, (2) the dynamic succession of the bacterial community, and (3) the reduction of potential pathogens during large-scale silo reactor composting in industrialized pig farms in China. The research provides evidence and guidance for regulating the full-scale composting process and improving compost quality and efficiency.

## 2 Materials and methods

### 2.1 Raw materials and experiment design

Two full-scale composting experiments were conducted in Sanmenxia City, Henan Province (112.30°E, 22.18°N), and Jiangmen City, Guangdong Province (111.12°E, 34.47°N). Composting was conducted in full-scale reactors (90 m^3^ in volume) that were cylindrical vessels with force aeration systems (Zhang et al., 2016). The wall of these reactors was designed as three layers, with one layer of thermal insulation material in the middle of two layers of stainless steel materials. There are air pumps at the bottom of the reactors to aerate the compost. The aeration and turning parameters in large-scale silo reactors was optimized lasting 2 consecutive months (Zeng et al., 2022). The best-optimized experiments lasting for 9 days were chosen for further comparison.

The raw materials were swine manure and carcasses from the composting factory in Sanmenxia City, Henan Province, and the treatment was named “HN” after the location. The other group from the composting factory of Jiangmen City, Guangdong Province, was named “GD,” whose raw materials were swine manure, carcasses, and mature compost. The properties of compost feedstocks in GD and HN are shown in Supplementary Table S1.

Composting samples were collected daily from days 1 to 9. Considering the effect of different depths in the large-scale silo reactors, samples were taken from five different layers on the 0.5, 1.0, 1.5, 2.0, and 3.0 m from top to bottom, named L1, L2, L3, L4, and L5, respectively (Supplementary Figure S1). Sampling was repeated at five depths by the 5-point sampling method. To ensure the representativeness of the samples, the sample was conducted three times at 15-min intervals by plotting sampling equipment and then mixed into one sample. The dry and fresh samples were stored separately at room temperature and −80°C for measurement.

### 2.2 Analytical methods

The temperature of the compost was detected by temperature sensors (PT100, United States) at five different levels (L1, L2, L3, L4, and L5), simultaneously with sampling. The pH was measured in water suspension (1:10, w/v) of compost with a pH meter (PHS-3C, China) (Liu et al., 2024). Total nitrogen (TN), total organic matter (TOC), and moisture content were measured according to the Chinese National Standard (NY/T 525–2021). Ammonium nitrogen (NH_4_^+^-N) was extracted from a 12 g fresh compost sample using a 1:10 ratio of soil to 0.01 mol L^−1^ of CaCl_2_ solution and then analyzed using continuous flow analysis (SEAL Analytical XY-2Sampler, England). Electrical conductivity (EC) was measured in water suspension (1:10, w/v) of compost with an EC meter (DDS-307A, China) (Liu et al., 2023). The seed germination index was measured following the method described by the appendix of the China Industry Standard (NY/T 3442–2019), where freshwater extraction of compost (1:10, w/v) was added to Petri dishes containing cucumber seeds.

Total DNAs of composting fresh samples were extracted using a Soil Fast DNA Spin Kit (MP Biomedicals, Santa Ana, Carlsbad, CA, United States). The 16S rRNA gene fragments were amplified with the universal primers 515F (5′-GTGCCAGCMGCCGCGGTAA-3′) and 806R (5′-GGACTACVSGGGTATCTAAT-3′) fused with a 12 nt unique barcode. Gel-purified PCR products were mixed with equal molar following the standard operating procedures of the Illumina MiSeq platform, and the platform of HiSeq2500 was used for sequencing (Zhang et al., 2021). All sequences were submitted to the NCBI (PRJNA783746). All sequence reads were assigned to each sample based on primer and barcodes, and the technical regions were trimmed for the following analyses. High-quality sequences (length > 300 bp, without ambiguous base ‘N,’ and average base quality score > 30) were used. Generation of the taxonomic OTU was performed as previously described (Wang et al., 2020).

### 2.3 Statistical analysis

The data were shown by the mean ± standard deviation. Figures were prepared using Origin 2020 (Origin Lab Corporation, United States). Shannon Wiener and Pielou’s evenness indices were calculated in a manner similar to rarefaction analysis, according to Wei et al. (2020). The plotting of the bacterial community was analyzed by R 3.1.21, and these tools were implemented into a galaxy instance^1^. Correlation analysis and redundancy analysis (RDA) with a forward selection were carried out using the I-Sanger platform^2^ provided by Majorbio Bio-Pharm Technology Co. Ltd. (Shanghai, China) (Sheng et al., 2021). To identify potential pathogens in the dataset, a standalone BLASTN analysis against the curate PATRIC^3^ database was conducted, according to Wang et al. (2020).

## 3 Results and discussion

### 3.1 Change in physicochemical properties

**Figure 1:**
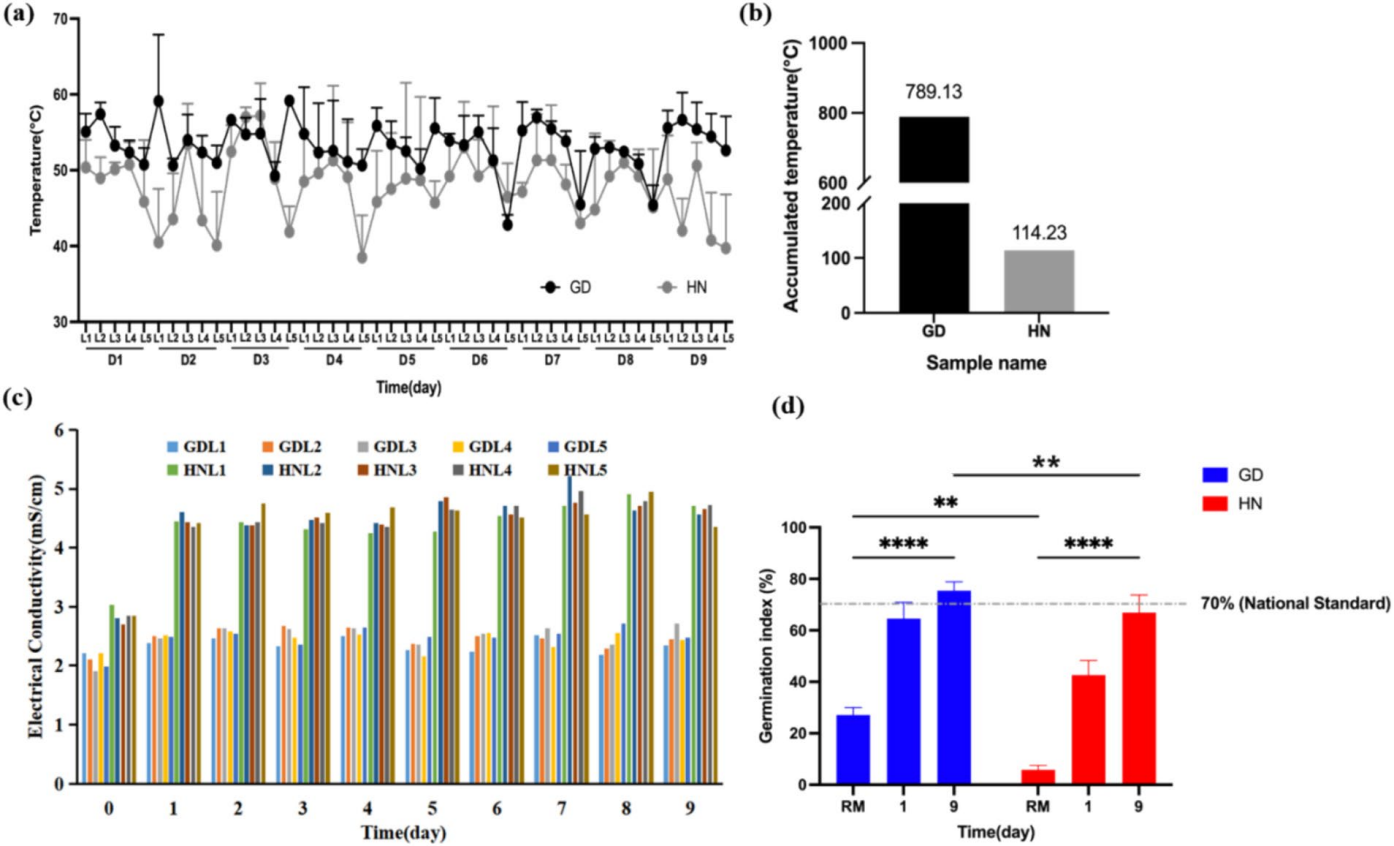
Variations of main physicochemical parameters in different treatments. (a) Temperature, (b) accumulated temperature, (c) electrical conductivity (EC), and (d) germination index (GI). RM, raw materials. Samples at 0.5 m, 1.0, 1.5, 2.0, and 3.0 m from the top to the bottom were named L1, L2, L3, L4, and L5, respectively. **p ≤ 0.01 for significance; ****p ≤ 0.0001.

Temperature reflected the microbial activity during the composting process, which is mainly separated into three stages: mesophilic, thermophilic, and cooling (Liu et al., 2020). As shown in Figure 1a, a similar trend of temperature curve was presented in two treatments, but the temperature in composting of GD was higher than that of HN. Since day 1, the temperature of all depths in reactors except the bottom level (L5, 3.0 m) was almost over 50°C in GD treatment and maintained a high temperature during the whole process in silo reactors, indicating that the compost was at the thermophilic phase (Kong et al., 2023). However, in the HN treatment, the temperature of all depths except the bottom level (L5, 3.0 m) was approximately 50°C with large fluctuations. In addition, the accumulated temperature for thermophilic stage (>55°C) in GD treatment (789.13°C) was higher than in HN treatment (114.23°C), with almost seven times (Figure 1b). Mature compost addition could alter the physical properties of the composting system, such as porosity, which may lead to increased oxygen content and utilization efficiency, thereby avoiding the anaerobic environment, facilitating the decomposition of organic matter, and promoting higher heat generation. In addition, the endogenous microorganisms of mature compost may show higher adaptability compared with indigenous microorganisms naturally, improving microbial richness and evenness. The results were consistent with the finding of Li et al. (2022), who found the positive effect of mature compost on temperature enhancement and antibiotic resistance gene reduction in food waste composting. These findings suggested that silo composting with mature compost is more effective in promoting harmlessness through higher temperatures, which can help in the eradication of pathogenic bacteria, weed seeds, and other harmful organisms (Wang et al., 2022).

The initial pH value of GD and HN was approximately 7.5 and then stabilized at 8.5–9.0 in 9 consecutive days of monitoring (Supplementary Figure S2). The pH of GD was a little higher compared to that of HN. Moisture content was decreased rapidly in the two treatments, which declined by 36.78 and 36.73% in silo composting reactors of GD and HN, respectively (Supplementary Figure S2). The *C*/*N* decreased slowly after the reactor composting process, which reduced from 12.68 to 10.35 in GD and from 10.19 to 9.50 in HN, respectively (Supplementary Figure S2). Moreover, there was more ammonium nitrogen reservation in GD compared to HN (Supplementary Figure S2), suggesting that mixing mature compost into raw materials in large-scale reactor composting helps to reduce nitrogen loss (Manu et al., 2022). The EC value of HN was significantly higher for 9 days than that in GD, and the final EC value was 2.48 mS/cm in GD treatment and 4.60 mS/cm in HN treatment (Figure 1c). Therefore, the addition of mature compost was beneficial for meeting the standards (<4 mS/cm) (Li et al., 2023). The germination index (GI) is one of the key indicators to reflect composting maturation (Kong et al., 2023). As shown in Figure 1d, the GI of GD treatment was significantly higher than HN treatment (*p* < 0.01) on day 1 due to adding mature compost. During the 9-day fermentation, the GI of these treatments was increased significantly (*p* < 0.001). At the end of the experiments, the GI of GD and HN treatment was 75.43 and 66.95%, respectively (Wang et al., 2022).

The above results suggested that adding mature compost can improve the compost structure and reduce anaerobic zones, promoting material degradation, which leads to relatively higher temperature, pH, and ammonium nitrogen. Meanwhile, moisture content was mainly influenced by aeration rate, and the two treatment results were similar. On the other hand, the HN treatment likely underwent facultative aerobic fermentation, with slower degradation and more small molecular substances remaining, whereas in the GD treatment, the degraded organic acid small molecules were quickly utilized or converted into gasses, resulting in a lower EC. Therefore, mature compost addition in swine manure and carcasses composting in the large-scale silo reactors increased the safety level of compost products and improved the fermentation efficiency.

### 3.2 Dynamics of bacterial community

**Figure 2:**
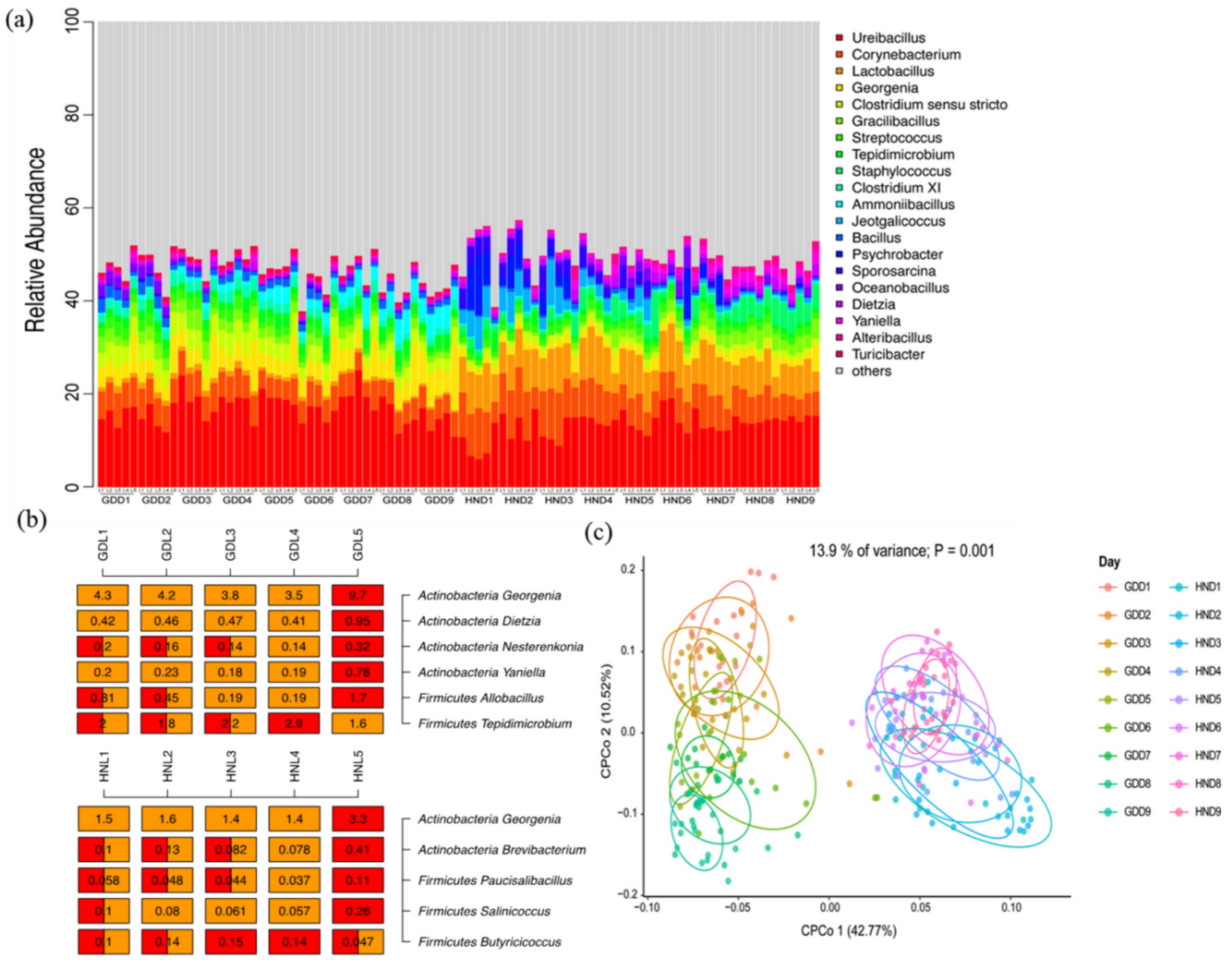
Variations in bacterial community diversity and composition during composting. (a) Relative abundance of the dominant genus of composting at the temporal and spatial scales in GD and HN, (b) heatmap of the genus in GD and HN, (c) constrained principal co-ordinate analysis of the bacterial community in composting in GD and HN. D1, D2, …, D9 mean the first day to the ninth day of composting, respectively.

Altogether, 2,828,472 high-quality reads were acquired based on high-throughput sequencing of 16S *rRNA* analysis. Most sequences were affiliated with Firmicutes and Actinobacteria, which were in agreement with previous reports (Wang et al., 2020). The elevated temperature conditions have favored the selection of Firmicutes specialized in thriving in high-temperature environments (Ma et al., 2019). The dominated genera in composting of HN were *Ureibacillus, Lactobacillus*, *Corynebacterium*, *Staphylococcus*, and *Jeotgalicoccus*, and most of the genera in GD belonged to *Ureibacillus, Corynebacterium*, *Georgenia*, *Clostridium sensu stricto*, and *Ammoniibacillus* (Figure 2a), suggesting that the addition of mature compost could change the composition of bacterial community, similar to the effect of inoculation (Wu et al., 2024). The relative abundance of *Ureibacillus* was 16.82% on average, significantly higher than that in HN. Studies suggest that mature compost and exposure to high temperatures can increase the occurrence of *Ureibacillus*, enhancing metabolic pathways for amino acids and carbohydrates and facilitating the breakdown of organic substances (Wang et al., 2022). Considering that *Ureibacillus* belonging to Firmicutes was related to organic matter degradation and can form spores to survive in high temperatures (Wang et al., 2022), its dominance may help to the harmlessness and maturation level of composting by increasing thermophilic temperature in reactors. The relative abundance of *Lactobacillus* in HN was significantly higher than that in GD. During the 9-day continuous composting of GD, the relative abundances of *Corynebacterium* and *Clostridium sensu stricto* decreased, whereas *Georgenia* and *Ammoniibacillus* increased significantly. Except for Staphylococcus, the relative abundance of the top five dominant genera of HN decreased (Figure 2a). ANOVA revealed that the bacterial alpha diversity in GD was higher than HN in composting (Supplementary Figure S3). As shown in Figure 2b, there was a significant difference in the bacterial community composition among five depths in the two treatments. The previous studies also found that the microbial community in composting may be affected by the depth and the bottom aeration strategy of large-scale reactors (Zhang et al., 2021). Constrained principal co-ordinate analysis indicated that the bacterial community from GD mainly clustered together and separated from that in HN, suggesting that mature compost addition significantly affected microbiota in co-composting swine manure and carcasses (Figure 2c). Variance partitioning revealed a prominent effect of the mature compost addition on bacterial communities, explaining 13.9% of the variance (*p* = 0.001). Over time, bacterial communities of GD were different, suggesting that alterations in the environmental conditions and substrate composition affected bacterial community composition (Liu et al., 2022).

The above results demonstrate that mature compost addition into swine manure and carcasses could increase the relative abundance and the number of microbial taxa involved with organic matter degradation (e.g., *Ureibacillus*), providing an appropriate temperature and pH environment for driving composting maturity.

### 3.3 Relationship between physicochemical properties and microbial community

**Figure 3:**
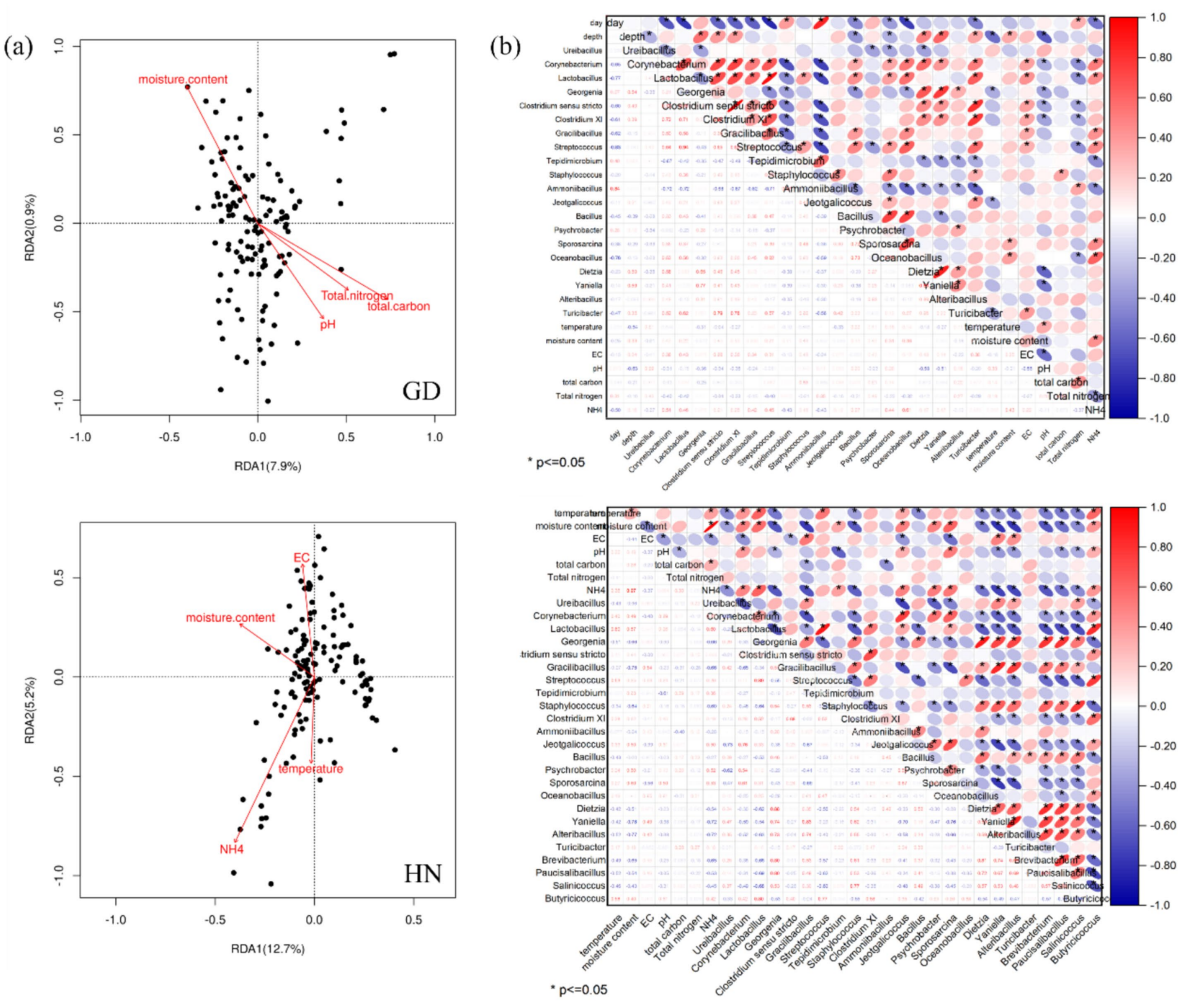
Relationship between physicochemical properties and microbial community in GD and HN. (a) RDA assessing the relationship between environmental parameters and microbial communities and (b) Spearman’s correlation heatmap of the correlation between environmental parameters and bacterial genera.

The RDA and Spearman’s correlation heatmap were used to analyze the relationships between key bacteria and physicochemical parameters (Figure 3). Redundancy analysis revealed that the beta diversity of bacterial communities of GD was significantly associated with moisture content, total organic matter content, total nitrogen content, and pH value, yet bacterial communities of HN were associated with moisture content, electrical conductivity, temperature, and ammonium nitrogen (Figure 3a). In addition, the correlation analysis showed that pH was negatively related to *Corynebacterium*, and the temperature was negatively associated with *Turicibacter, Jeotgalicoccus, and Georgenia* in GD. In HN, temperature showed a positive correlation with *Corynebacterium, Lactobacillus, Jeotgalicoccus*, and *Butyricicoccus*, and moisture had a negative correlation with *Ureibacillus, Georgenia, Staphylococcus, Dietzia*, *Yaniella*, *Alteribacillus*, and *Brevibacterium*. The content of NH_4_^+^-N had a positive correlation with *Corynebacterium*, *Lactobacillus*, and *Sporosarcina* in both GD and HN. *Ureibacillus, Georgenia, Gracilibacillus, Staphylococcus*, and *Dietzia* were significantly negatively associated with ammonia nitrogen (*p* < 0.05) in HN. These results were consistent with previous studies that environmental variables such as temperature and nitrogen are a part of the determinants for bacterial community succession during composting (Wang et al., 2013).

These results demonstrated that mature compost addition may change the moisture content, pH, and EC properties of reactor composting, which is conducive to the colonization and survival of thermophilic bacteria while increasing organic matter degradation and affecting ammonia nitrogen concentration. Total nitrogen also plays a crucial role in shaping bacterial community composition, and high temperatures and *Ammoniibacillus* influenced the utilization of NH_4_^+^-N and the release of NH_3_ (Liu et al., 2024). Moreover, the alterations in the relative abundance of other genera may be associated with the harmlessness level of composts, including *Georgenia, Jeotgalicoccus, Yaniella, Dietzia, Staphylococcus*, and *Staphylococcus*. There were more genera of the bacterial community significantly related to temperature in HN compared to GD, which was more suitable for bacterial proliferation and survival probability of potential pathogens in medium temperature (30–50°C).

### 3.4 Impact on potential bacterial pathogens

**Figure 4:**
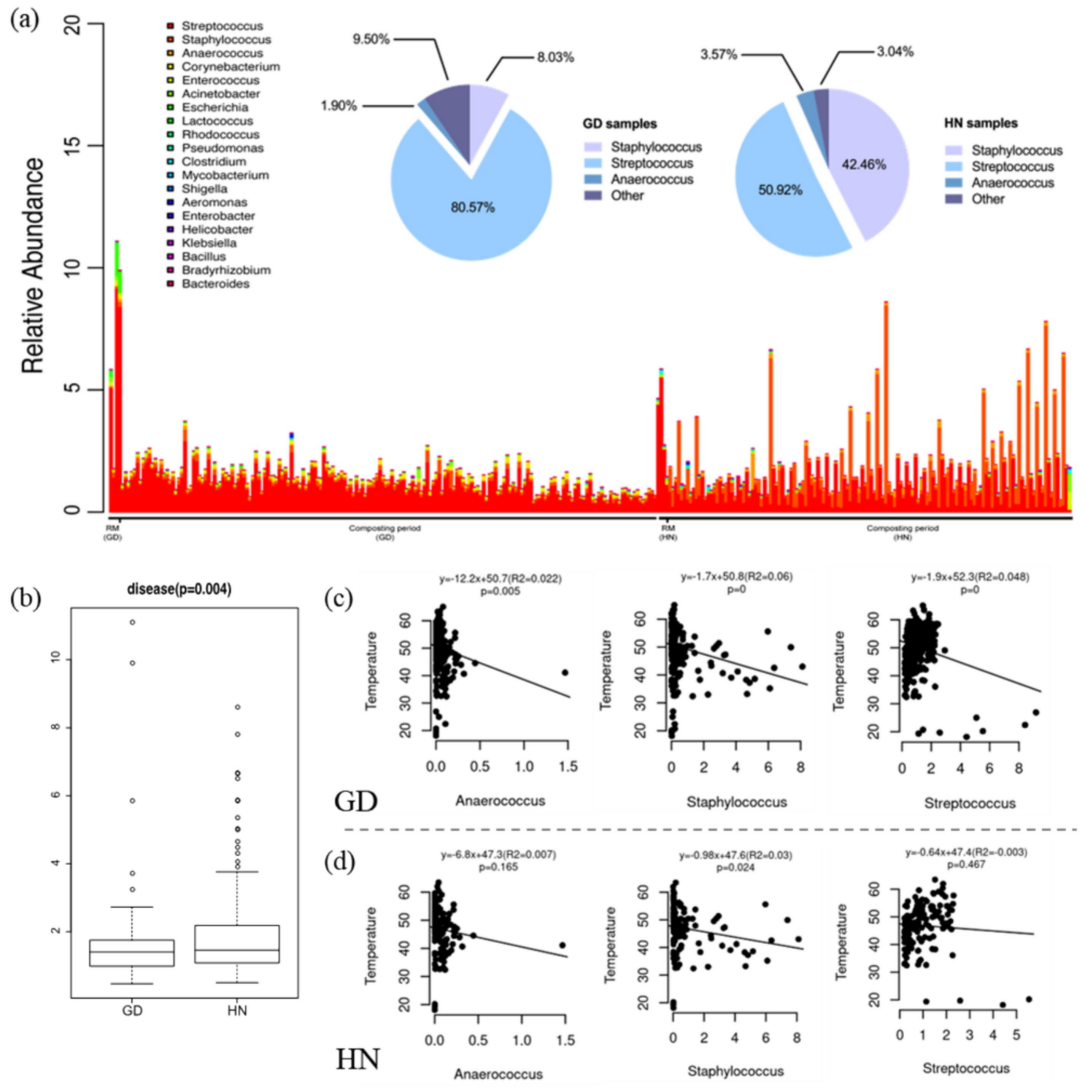
Potential bacterial pathogens in composting of GD and HN. (a) Relative abundance of potential bacterial pathogens in composting, (b) the mean ratio of potential pathogens in compost, (c) correlation of the main potential bacterial pathogens with temperature in GD, and (d) correlation of the main potential bacterial pathogens with temperature in HN. RM, raw materials.

The feces from intensive livestock farming contain a variety of pathogenic microbes that may contaminate soil, air, and water (Black et al., 2021). In composting, the thermophilic phase with over 50°C can help to kill pathogens (Fröschle et al., 2015); however, there is little information about the diversity and abundance of the potential pathogens in large-scale composting reactors. A standalone BLASTN analysis against the curated databases containing human and animal pathogens was conducted, which detected a total of 421 potential bacterial pathogens belonging to 39 genera (Figure 4a). The majority of them were affiliated with *Streptococcus*, *Staphylococcus*, *Anaerococcus*, *Corynebacterium*, etc., which were significantly negatively correlated with temperature. It has been reported that *Staphylococcus* and *Streptococcus* were opportunistic pathogens that were commonly detected in air and soil (Zhang et al., 2020). The relative abundance of the potential pathogen in the raw material of GD was higher than in HN (Figure 4a). After 9-day fermentation, the potential pathogen decreased from 7.16 to 0.77% in GD, and the relative abundance of the potential pathogen in HN ranged from 3.69 to 2.97%, which might be related to the lower accumulated temperature in HN than GD (Wang et al., 2020). It was found that the dominated genus of potential pathogens in GD was *Streptococcus*, accounting for 80.57%. In HN, *Streptococcus* and *Staphylococcus* constituted most of the potential pathogens, which accounted for 50.92 and 42.46%, respectively. The ratio of pathogens in the bacterial community in composting in HN was significantly higher than that in GD (*p* = 0.004) (Figure 4b). Interestingly, the relative abundance of these three genera was significantly negatively correlated with temperature in both GD (Figure 4c) and HN (Figure 4d), which indicated that the thermophilic environment was beneficial for killing the pathogens.

The above results indicated that there is a better harmlessness effect in GD compared to HN based on the change of temperature, GI, and the ratio of potential bacterial pathogens. Altogether, adding mature compost into raw materials (swine manure and carcasses) in continuously running large-scale silo reactors significantly improved the harmlessness level and humification degree of composting, which provides an effective and environmentally friendly scheme to deal with the main organic wastes in intensive pig farms.

## 4 Conclusion

This study conducted a comparative analysis of two continuously running large-scale silo reactor composting systems in intensive pig farms in HN and GD. The GD group with adding mature compost into swine manure and carcasses increased the highest temperature and had a 7-fold higher accumulated temperature compared to HN. Though there were different bacterial communities at different times and depths in silo reactor composting, the dominated genera were *Ureibacillus, Corynebacterium*, *Georgenia*, *Clostridium sensu stricto*, and *Ammoniibacillus*. Mature compost addition promoted the microbial genera involved with organic matter degradation and nitrogen conversion, such as *Ureibacillus*. The majority of potential pathogens were affiliated with *Streptococcus*, *Staphylococcus*, *Anaerococcus*, etc., which had significantly negative correlations with temperature. The relative abundance of potential pathogens in GD reduced to 0.77%, significantly lower than that (2.97%) in HN. Therefore, mature compost addition can enhance the harmlessness level.

Future studies should implement a systematic monitoring program for bacterial pathogens to assess the impact of different composting methods on the abundance and activity of potential pathogens. Investigating the mechanisms by which mature compost addition reduces pathogen levels will provide valuable insights for improving composting practices and ensuring food safety.

## Data Availability

The datasets presented in this study can be found in online repositories. The names of the repository/repositories and accession number(s) can be found in the article/Supplementary material.

## Footnotes

^1^ www.freebioinfo.org

^2^ https://www.majorbio.com/

^3^ www.patricbrc.org
