# Supplementary material for: Mature compost enhanced the harmlessness level in co-composting swine manure and carcasses in large-scale silo reactors
Source: Front Microbiol. 2024 Nov 13;15:1494332. doi: 10.3389/fmicb.2024.1494332 (PMC11599618; doi:10.3389/fmicb.2024.1494332)
Supplement: Supplementary file 1 [file Data_Sheet_1.docx]

**Supplementary Materials**

Supporting Information Includes:

- 1 table

- 3 figures

**Table** S1. The main characteristics of raw materials of composting

|  | **Total carbon (%)** | **Total nitrogen (%)** | **Moisture Content (%)** | **Electrical conductivity (mS/cm)** | **pH** |
| --- | --- | --- | --- | --- | --- |
| GD | 37.31 ± 1.19 | 2.95 ± 0.15 | 75.39 ± 3.21 | 2.08 ± 0.14 | 7.45 ± 0.26 |
| HN | 41.12 ± 0.84 | 4.05 ± 0.20 | 76.09 ± 1.31 | 2.84 ± 0.12 | 7.26 ± 0.17 |
| Mature compost | 35.07 ± 2.56 | 3.56 ± 0.59 | 38.85 ± 1.20 | 2.48 ± 0.30 | 8.75 ± 0.08 |

The raw materials including swine manure and carcasses in Sanmenxia were named as “HN” and those in Jiangmen named “GD”.


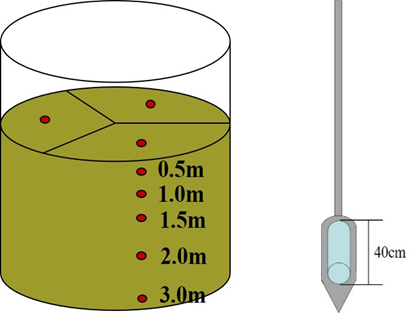


**Fig**.S1 Sampling scheme in the silo reactor in the composting process. The red points represent the sampling points and the diagram on the right shows the sampler used in the experiments.


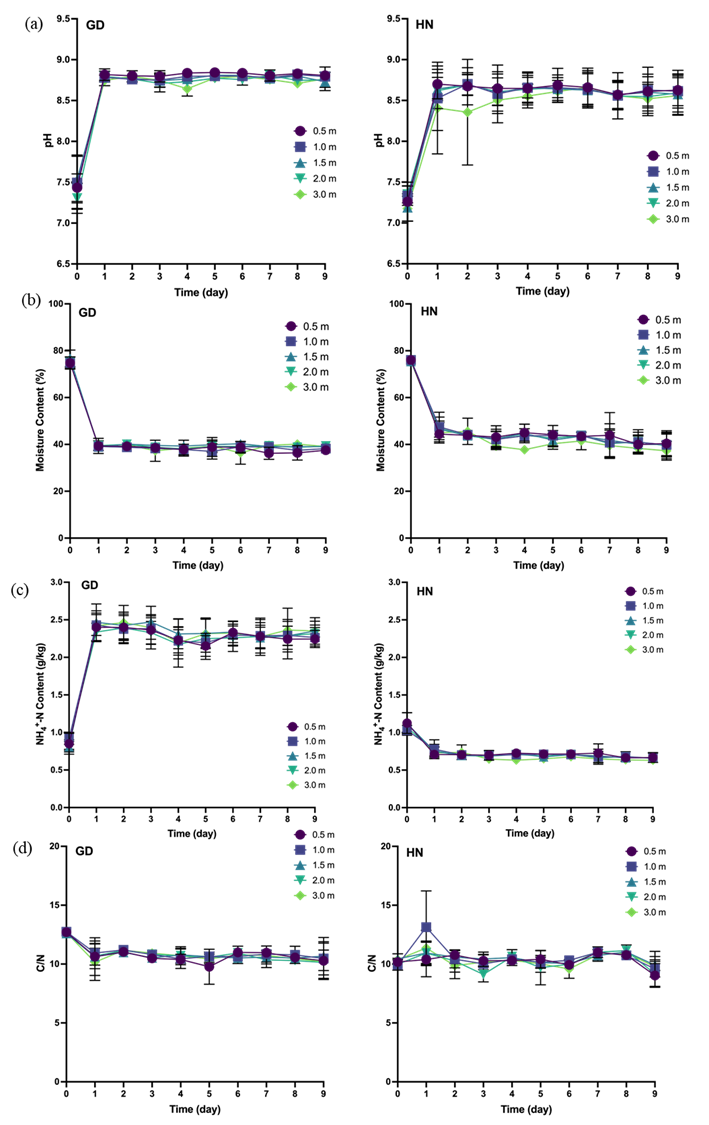


**Fig**.S2 Variations of physicochemical parameters and maturity indicators at different sampling depths in different treatments (GD and HN) during 9-day composting including (a) pH, (b) moisture content, (c) NH_4_^+^-N content, (d) carbon-to-nitrogen ratio (C/N).


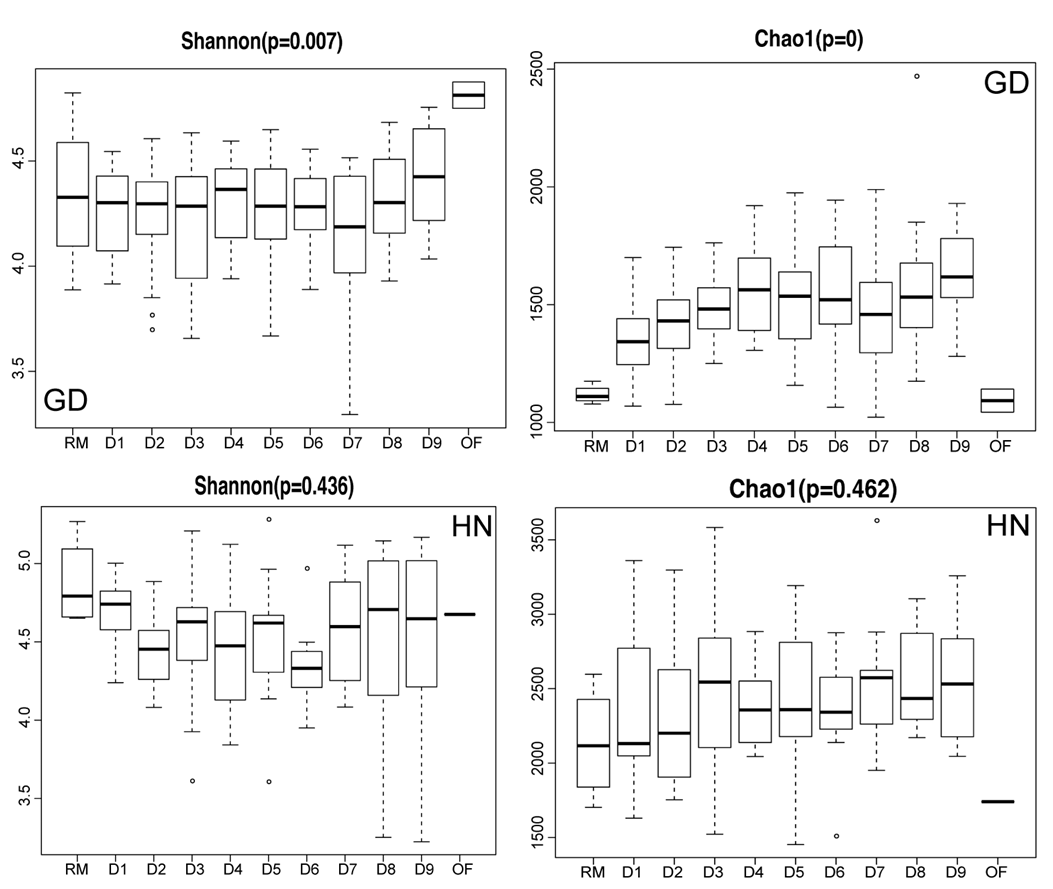


**Fig**.S3 Variations of Alpha Diversity of bacterial community in composting of GD and HN. RM, raw materials. D1, D2, …, D9 mean the first day to ninth day of composting, respectively. OF, organic fertilizer.
